# The influence of leader-subordinate emotional intelligence congruence on the flow experience: evidence from the liquor manufacturing industry

**DOI:** 10.3389/fpsyg.2024.1324721

**Published:** 2024-04-24

**Authors:** Zhuoran Lu

**Affiliations:** ^1^School of Management, Xi’an Jiaotong University, Xi’an, China; ^2^Business Research Laboratory, Xifeng Liquor Group Co. Ltd., Xi’an, China

**Keywords:** PE-fit, polynomial regression, leader-subordinate EI congruence, LMX, flow experience

## Abstract

Previous research has provided evidence supporting a positive correlation between emotional intelligence (EI) and flow. However, intriguing findings in the liquor manufacturing industry prompt me to consider the necessity of examining the effects of leader-subordinate EI congruence on flow. Therefore, this article begins with two questions: First, what is the impact of leader-subordinate EI congruence on flow? Second, do two distinct congruent scenarios (i.e., high-high and low-low) exhibit significant nuances that differentially influence flow? To answer these questions, this study utilizes polynomial regression and three-dimensional response surface analysis methods. A multi-source and three-phase investigation method was used to collect data from 279 subordinates and 56 leaders in the liquor manufacturing industry. Based on the person-environment (PE) fit theory, this study investigates the influence of leader-subordinate EI congruence on flow while considering leader-member exchange (LMX) as a mediator in these processes. The current study not only demonstrates a positive relationship between leader-subordinate EI congruence and LMX, but also reveals that a high-high EI matching pattern can enhance the favorable dynamics of congruence and yield higher LMX compared to a low-low matching pattern. Furthermore, this study identifies LMX as a mediator in the positive relationship between leader-subordinate EI congruence and flow. Additionally, although the incongruent scenarios are beyond the scope of this study, the findings demonstrate that a high EI subordinate paired with a low EI leader has a more detrimental impact on the LMX than a low EI subordinate paired with a high EI leader. Theoretical and practical implications are discussed.

## 1 Introduction

In order to promote active participation and enjoyment at work among subordinates, scholars have shown great enthusiasm for the proliferation of studies on positive organizational behavior (POB) over the past decade (Luthans, 2002; Salanova et al., 2006). Flow, as a core concept of POB, refers to a psychological state experienced by individuals when they are fully immersed in an activity and oblivious to everything else (Csikszentmihalyi and Rathunde, 1993). Subordinates who experience flow report higher levels of enjoyment, initiative, and performance while experiencing less psychological discomfort (Aust et al., 2022). Therefore, cultivating a state of flow is crucial for both subordinates and organizations.

Emotional intelligence (EI) encompasses the ability to identify, comprehend, utilize, and regulate emotions (Mayer and Salovey, 1997). Scholars have underscored the significance of subordinates’ EI on their organizational behavior within the manufacturing and retail industry (Xie, 2021; Jain et al., 2023). Therefore, in order to achieve a state of flow, subordinates in liquor manufacturing industry may need to effectively employ EI in regulating their own emotions and accurately perceiving the emotions of others (Xie, 2021). However, intriguing findings in the liquor manufacturing industry prompt me to consider that the relationship between EI and flow in real-world situations may be more complex and require further exploration of leader-subordinate EI congruence. Specifically, recognizing the crucial role of EI in effectively facilitating subordinates’ flow within a similar industry (Xie et al., 2021), and acknowledging that “finding flow is the holy grail of business success for individual employees, teams, and companies” (p. 8) (Van den Hout and Davis, 2019), a renowned Chinese liquor manufacturing group has implemented an annual training program encompassing a diverse range of EI training activities specifically tailored for leaders and subordinates. Despite the steady increase in leaders’ and subordinates’ EI, many subordinates still perceive limited progress or even regression in their state of flow. This is evident from slight improvements or declines in efficiency, motivation, and joy. In investigating the root cause of this phenomenon, the manufacturing group identified that leader-subordinate EI interaction may capture the overall impact of EI and play a significant role in influencing the focal subordinate’s state of flow. Exploring the impact of leader-subordinate EI congruence on flow may provide further insights into explaining this phenomenon, as the benefits of EI are acquired through interpersonal interactions among stakeholders in the workplace (Mayer et al., 2008). Furthermore, one of the fundamental principles of person-environment fit theory suggests that the person and the environment together predict human behavior better than each of them does separately (Van Vianen, 2018). Surprisingly, there has been no prior investigation into the factors that influence flow in terms of the interaction between personal and organizational attributes (Peifer and Wolters, 2021). Therefore, drawing on the aforementioned theoretical and practical perspectives, this study aims to investigate 279 subordinates and 56 leaders across four subsidiaries within this liquor manufacturing group. The study aims to investigate two important questions regarding leader-subordinate EI congruence: First, what is the influence of leader-subordinate EI congruent interaction on flow? Second, do high-high and low-low congruent scenarios exhibit crucial nuances that differentially influence flow?

According to PE fit theory, it is probable that a subordinate’s experience of flow does not depend solely on their individual EI; instead, congruent EI dynamics between the focal leader and subordinate also play a significant role in shaping this outcome. As a result, exploring the impact of EI congruence between two loci (i.e., leader and subordinate) provides a fertile ground for examining the influence of interaction effects on flow. Furthermore, drawing on the PE fit theory, leaders may establish and maintain different levels of LMX with subordinates in their dyadic EI interactions based on the level of EI fit. This aligns with the leader-member exchange theory of leadership, which emphasizes the significance of emotional exchanges and mutual emotional fits in fostering high-quality development of LMX relationships (Graen and Uhl-Bien, 1995). The literature has emphasized the pivotal role of LMX in shaping subordinates’ desirable work behaviors in terms of cognitive and affective states (Mueller and Lee, 2002; Radstaak and Hennes, 2017; Estiri et al., 2018). Therefore, based on the aforementioned rationale, I expect LMX to serve as a crucial dyadic characteristic mediating the relationship between leader-subordinate EI congruence and flow. Furthermore, in response to the scholarly demand for investigating detailed information on interaction effects in organizational settings (Edwards and Parry, 1993), this study utilized polynomial regression and three-dimensional response surface analysis methodologies to evaluate all proposed hypotheses and address the two questions raised in the previous paragraph based on two EI congruent scenarios (high-high, low-low).

This study is expected to make theoretical contributions in the following ways. First, the present study contribute to the existing literature on PE fit. (a) This study recognizes that individuals do not exist in isolation but rather exchange within a social context where leaders play a crucial role. That said, a leader and a subordinate who share similar characteristics, particularly EI, exchange more effectively and therefore develop desirable flow experience. By exploring this novel perspective, I hope to shed light on a more comprehensive understanding of the dynamics of PE fit in organizational settings. (b) Some scholars in the field of PE fit argue that regardless of absolute levels, PE fit produces identical outcomes; however, others express doubts regarding this proposition which necessitate further verification (p. 216) (Edwards, 2008). This study offers valuable insights into the asymmetric effects of PE fit and misfit based on four scenarios (high-high, low-low, high-low, and low-high). Second, this study makes two contributions to the literature on flow. (a) This study provides the initial empirical investigation into the influence of interaction effects on flow, as previous research on this subject has primarily been theoretical: “…a holistic fit of both the relevant attributes of an individual with the attributes of the job/task sphere and the attributes of the organizational/social sphere should provide the greatest flow potential…” “…to our best knowledge, the interaction of personal and organizational attributes has not yet been investigated and should clearly receive research attention in the future…” (P. 307) (Peifer and Wolters, 2021). (b) This study enhances understanding of the locus of flow. While knowledge of specific determinants is important, I suggest that understanding the influence of interactions between different loci (i.e., leaders and subordinates) helps frame the consequences of determinants within a broader framework where flow emerges. By focusing on locus interactions rather than individual determinants, this study is able to compare the influence exerted by all underlying characteristics at each locus. This approach facilitates the identification of influential factors in flow research at deeper levels, thereby highlighting promising areas for further study. In summary, this study contributes to the advancement of flow theory within an extensive and inclusive framework (Peifer and Wolters, 2021; Peifer et al., 2022). Third, this study contributes novel insights to the existing literature on EI and LMX by utilizing polynomial regression and three-dimensional response surface analysis methodologies. This approach provides a novel lens for exploring potential outcomes of EI within leader-subordinate dynamics, such as LMX, thereby enhancing theoretical understanding of the nuanced variations in the relationship between EI and LMX (Lopes et al., 2004; Sy et al., 2006). In addition, this study also aims to offer practical suggestions for organizations in effectively promoting flow within the workplace.

## 2 Hypotheses

### 2.1 Leader-subordinate EI congruence and LMX

The present study adopts the PE-fit theory as the overarching theoretical framework. The fundamental assumption of the PE-fit theory is grounded in the similarity-attraction paradigm, positing that a higher degree of similarities can foster enhanced interpersonal attraction and harmony between two parties (Byrne, 1971). PE-fit offers an effective framework for analyzing the dynamics between leaders and subordinates, thereby facilitating a comprehensive understanding of subordinates attitudes and behaviors (van Vianen, 2000; van Vianen et al., 2011). According to the concept of PE-fit, leaders and subordinates seek to confirm their cognition and view of the world. When there is congruence between a leader and a subordinate, they establish a shared understanding, experience a sense of connection, and hold similar expectations regarding behavioral norms (Kristof-Brown et al., 2005; Uhl-Bien, 2006). The aforementioned similarities may significantly impact the reciprocal evaluations and establishment of relationships between the focal leader and subordinate (Kristof-Brown and Guay, 2011).

The concept of LMX represents a reciprocal exchange process that evolves through the progressive development of stronger interpersonal affect and enhanced role definition (Dansereau et al., 1975; Dienesch and Liden, 1986). According to the PE-fit theory, the presence of greater similarities can facilitate enhanced interpersonal attraction, harmony, and exchange between the focal leader and subordinate. Consequently, effective interpersonal communication processes and high-quality exchange relationships are more likely to occur in the leader-subordinate EI congruent dynamics (Byrne, 1971). Similarly, leaders and subordinates may develop a stronger sense of interpersonal closeness, emotional attachment, and interactive engagement towards individuals whose EI align with their own (Kristof-Brown et al., 2005). In addition, if a supervisor and a subordinate exhibit a congruent inclination towards effectively managing and leveraging emotions in their workplace interactions, this similarity may serve to validate and reinforce their self-perceptions regarding emotional utilization and role definition, ultimately leading to an enhanced quality of LMX. This aligns with the broaden-and-build effect (Fredrickson, 2001), which posits that a shared capacity to recognize, comprehend, utilize, and regulate emotions within the leader-subordinate dynamics may facilitate the development of upward spirals towards accumulating enduring psychological and social resources for both the focal leader and subordinate. This, in turn, enhances their appreciation and engagement in reciprocal exchange processes within the leader-subordinate dynamics. To summarize, the aforementioned findings and arguments collectively support the formulation of the following hypothesis:

*H1*: Leader-subordinate EI congruence is positive related to LMX.

### 2.2 EI congruence in high versus low

The rationale behind the examination of leader-subordinate EI congruence aligns with existing PE-fit research, which suggests that leader-subordinate congruence generally yields positive outcome (Kristof-Brown et al., 2005). The aforementioned assumption, however, may obscure crucial nuances in the leader-subordinate EI dynamics. To attain a more comprehensive understanding of EI within leader-subordinate relationships, it may be imperative to acknowledge that the impacts of congruence are not equally positive. Specifically, the leader-subordinate EI congruence can be further categorized into high-high and low-low EI congruence, respectively. In comparison to the low-low EI congruence scenario, the high-high scenario is likely to enhance favorable dynamics of congruence.

According to Bar-On model of emotional-social intelligence (ESI), EI encompasses the capacity for self-awareness, self-understanding, and self-expression; the ability to perceive, comprehend, and engage with others; proficiency in managing intense emotions and regulating impulsive behaviors; as well as adaptability in navigating personal or social challenges and resolving problems (Bar-On, 2006). Therefore, individuals with higher EI (compared to those with low EI) demonstrate a greater capacity in facilitating self-awareness, understanding of others, effective expression, and adept handling of social demands. That is to say, when there is a high-high level of leader-subordinate EI congruence matching (compared to low-low matching), leader-subordinate dynamics may be more interactive, cooperative, and effective. The occurrence of more LMX is therefore expected in a high-high leader-subordinate EI congruence matching pattern compared to a low-low matching pattern. Specifically, Goleman (2003) emphasized that leaders with high EI exhibit superior abilities in managing relationships. They possess a deeper understanding of their employees and cultivate a more harmonious and approachable work environment (Drigas and Papoutsi, 2019). Likewise, subordinates with high EI exhibit enhanced social awareness and improved social management skills, enabling them to integrate more effectively into their team (Drigas and Papoutsi, 2019). Consequently, high-high leader-subordinate EI congruence matching may facilitates stronger LMX. To be more precise, leaders with high EI will be able to transmit their thoughtfulness to their subordinates making them feel more efficient, happier and satisfied at work (Drigas and Papoutsi, 2019). High EI subordinates are more skilled at managing their relationships with their leaders by excelling in the practice of “managing upward” (Sy et al., 2006). Therefore, the high-high scenario has the potential to enhance positive dynamics of congruence through the facilitation of positive emotions and upward spirals within the organization (Fredrickson, 2003). This, in turn, can lead to more effective interpersonal communication processes and higher quality exchange relationships between the focal leader and subordinate. The following hypothesis is proposed in summary:

*H2*: In cases of leader-subordinate EI congruence, a high-high EI matching pattern between leaders and subordinates leads to higher LMX than a low-low matching pattern.

### 2.3 The mediating effect of LMX

LMX focuses on the leader-subordinate dyadic relationship at the organizational level, placing emphasis on the quality of interaction between these parties. LMX posits that cultivating strong emotional connections between a leader and a subordinate may facilitate various desirable work behaviors in terms of cognitive and affective states (Dansereau et al., 1975). Thus, LMX, which involves high-quality interpersonal relationship between a leader and a subordinate, presents a potential avenue for enhancing the focal subordinate’s flow experience.

Specifically, the leader in high quality LMX provides the focal subordinate a wide range of resources, encompassing both tangible and intangible assets such as information, feedback, social support, and valuable assignments (Martin et al., 2010; Schyns and Day, 2010). Given that subordinates experience flow when their basic needs are satisfied (Bakker and van Woerkom, 2017), a high quality LMX may stimulate the focal subordinate with the necessary energy and resources to attain this optimal psychological state. Moreover, in terms of the relationship between LMX and flow experience, the presence of a clearly defined and precise purpose, constructive feedback, and effective open communication channels in high-quality LMX may correspond to an increased level of flow experience for focal subordinate (Nakamura and Csikszentmihalyi, 2005). Likewise, the findings of a study conducted by Bakker (2005) indicate that music teachers who receive high levels of social support and feedback are more likely to experience flow. In summary, the present study posits that LMX possesses the motivational capacity to enhance focal subordinate’s flow experience.

Based on the combination of the impact of leader-subordinate EI congruence on LMX and the influence of LMX on flow experience, the following hypothesis is proposed:

*H3*: LMX mediates the positive relationship between leader-subordinate EI congruence and flow experience.

## 3 Materials and methods

### 3.1 Participants and procedures

This study collected data from four subsidiaries of a renowned liquor manufacturing group in China, situated within a high-tech industrial park in western China. These subsidiaries encompass the interconnected sectors of liquor production, intelligent manufacturing, marketing, and retail. The manufacturing group implements a system that promotes the integration and efficiency of the liquor industry chain by facilitating mutual mobility between leaders and subordinates across its four subsidiaries, while maintaining standardized leader-subordinate relationships and distinct divisions of labor among subordinates. Prior to commencing the research, I requested the human resources department to extend invitations to eligible subordinates who have maintained their current positions for a minimum duration of 6 months, thereby ensuring a comprehensive understanding of their work and fostering stable leader-subordinate relationships. The subordinate information provided by the human resource department was analyzed, and no significant similarities were identified. In addition, we pre-surveyed 61 subordinates and 12 leaders randomly to assess the clarity and reasonableness of the questionnaire. The analysis of cross-sectional data confirmed the reliability and validity of questionnaire. In summary, this study enhances confidence in the representativeness and applicability of research tools and samples.

In the formal surveys, a multi-source and three-phase investigation approach was implemented to mitigate potential common source bias. The questionnaire was assigned code numbers for the purpose of matching and distributed in paper format. It was sealed and collected after each round of survey. The human resources department provided the subordinates’ demographic information prior to the formal survey initiation (time 0). During the first survey phase (time 1), I distributed around 430 questionnaires to leaders and subordinates for evaluating their EI. After eliminating inconsistent or incomplete questionnaires and excluding those from teams with less than three members (Carter and Mossholder, 2015), 346 subordinates and 68 leaders responses were retained following code matching. One month later, subordinates who had completed the first round were invited to participate in the second phase of survey (time 2) and their LMX was measured. Due to the absence of certain subordinates who were either on business trips or had resigned, I implemented the same screening process for questionnaires as in the initial phase and collected a total of 316 questionnaires from subordinates and 63 questionnaires from leaders. After an additional month, subordinates who had completed the second round were invited to participate in the third survey phase (time 3) and have their flow experience measured. After conducting the same screening process used in both the first and second survey phases, I collected 279 subordinate questionnaires and 56 leader questionnaires, resulting in response rates of 80.63 and 82.35%, respectively. The average team size was 4.98 subordinates.

The demographic characteristics of leaders in the paired samples were as follows: 64.29% were male and 35.71% were female; their average age was 36.12 years with a standard deviation of 4.36, and their average years of education was 17.12 with a standard deviation of 2.06. The demographic profile of subordinates was as follows: 59.86% male, 40.14% female, with an average age of 30.66 years (standard deviation = 4.32), an average educational attainment of 15.13 years (standard deviation = 2.62), and an average tenure with the supervisor of 3.26 years (standard deviation = 1.61).

### 3.2 Measures

This study used a 16-item scale by Wong and Law (2017) to measure Leaders’ and subordinates’ EI. The sample item included “I really understand what I feel.” The Cronbach’s α was 0.94 for leader EI and 0.94 for subordinate EI.

LMX was measured with 7-items scale by Graen and Uhl-Bien (1995). Subordinates were asked to respond to items such as: “How would you characterize your working relationship with your leader.” The Cronbach’s α was 0.87.

This study used Bakker (2008) 13-item scale to measure flow experience. The sample item included “when I work, I lose track of time.” The Cronbach’s α for this scale was 0.92.

Previous research suggests that individual EI and leader-subordinate congruence may be related to similarities in the demographic characteristics such as gender, age, and education level (Phillips and Bedeian, 1994; Cole et al., 2013; Matta et al., 2015; Jena and Goyal, 2022). Therefore, I controlled these variables in this study. In addition, consistent with Wong and Law (2017) and Cole et al. (2013), the dyadic tenure of each leader and subordinate were also controlled to exclude the potential familiarity effect.

To prevent central tendency bias, a six-point Likert scale (ranging from 1 for “strongly disagree” to 6 for “strongly agree”) was used to measure all variables because Chinese respondents tend to show a higher preference for the middle of the scale (Harzing, 2006; Xie et al., 2021). The back-translation method proposed by Brislin (1980) was used to translate the items from English into Chinese. The psychometric properties of all study measures used in this research have been demonstrated to be satisfactory within the Chinese organization’s context. In addition, an introductory letter was included at the beginning of the questionnaire to clarify that participants’ data would solely be used for research purposes and emphasize their right to withdraw from the study at anytime.

### 3.3 Analytical methods

The present study employs polynomial regression and three-dimensional response surface methodology to investigate the congruence effect of leader-subordinate EI (Edwards and Parry, 1993). The polynomial regression model, which predicts LMX using leader-subordinate EI, incorporates higher-order terms of both variables beyond their linear term (Cohen et al., 2010). In the polynomial regression equation, Y represents the dependent variable (LMX), while L and S represent leader and subordinate EI, respectively. To streamline the equation, I have excluded control variables from this equation while incorporating them into the analysis:


(1) $$Y=b_{0}+b_{1}\left(L\right)+b_{2}\left(S\right)+b_{3}\left(L^{2}\right)+b_{4}\left(L\times S\right)+b_{5}\left(S^{2}\right)+e$$

The regression coefficients are used to plot three-dimensional response surfaces, with L and S on the horizontal axes and Y on the vertical axis (Edwards and Parry, 1993). The plot’s horizontal plane consists of two lines: the congruence line (L = S) where leader-subordinate EI scores are equal, and the incongruence line (L = −S) indicating opposite signs but equal absolute values for leader EI and subordinate EI scores. The slope and curvature of the congruence line are calculated by adding specific coefficients from Eq. 1, namely b_1_ + b_2_ for slope and b_3_ + b_4_ + b_5_ for curvature (Edwards and Parry, 1993; Edwards and Cable, 2009). I will test hypotheses 1 and 2 by using characteristics of the response surface that provide evidence for the congruence effect. As for hypotheses 3, polynomial regression coefficients are used to establish a block variable for testing this mediating effect (Edwards and Cable, 2009).

## 4 Results

Table 1 presents descriptive statistics, internal consistency reliability, and Pearson correlation for the core variables. Leader EI is significantly positively correlated with subordinate EI (r = 0.30, *p* < 0.01). Subordinate EI is significantly positively correlated with LMX (r = 0.60, *p* < 0.01) and flow experience (r = 0.40, *p* < 0.01). LMX is significantly positively correlated with flow experience (r = 0.51, *p* < 0.01). The structural validity of the variables was assessed through confirmatory factor analysis (CFA) before testing hypothesized predictions. The results presented in Table 2 demonstrate that the four-factor model exhibits a good fit (*χ*^2^/df = 1.25, IFI = 0.96, PNFI = 0.79, RMSEA = 0.03, CFI = 0.96, TLI = 0.95). Furthermore, the goodness of fit for the four-factor model significantly surpasses that of the other four alternative models.

**Table 1:** Descriptive statistics and correlations.

Variables	M	S.D.	1	2	3	4
Organization-level variables						
Leader emotional intelligence	3.83	0.90	(0.94)			
Individual-level variables						
Subordinate emotional intelligence	3.78	0.92	0.30**	(0.94)		
LMX	3.63	1.01	0.10	0.60**	(0.87)	
Flow experience	3.70	0.96	0.30	0.40**	0.51**	(0.92)

*** *p* < 0.001, ** *p* < 0.01, * *p* < 0.05.

**Table 2:** Comparison of measurement models.

Model	*χ*^2^/df	IFI	PNFI	RMSEA	CFI	TLI
Single-factor model (LEI+SEI + LMX + FE)	4.33	0.47	0.39	0.10	0.47	0.45
Two-factor model (LEI+SEI + LMX, FE)	3.28	0.64	0.53	0.09	0.64	0.62
Three-factor model (LEI+SEI, LMX, FE)	2.93	0.69	0.57	0.08	0.69	0.68
Four-factor model (LEI, SEI, LMX, FE)	1.25	0.96	0.79	0.03	0.96	0.95

LEI, leader emotional intelligence; SEI, subordinate emotional intelligence; LMX, leader-member exchange; FE, flow experience.

**Figure 1:**
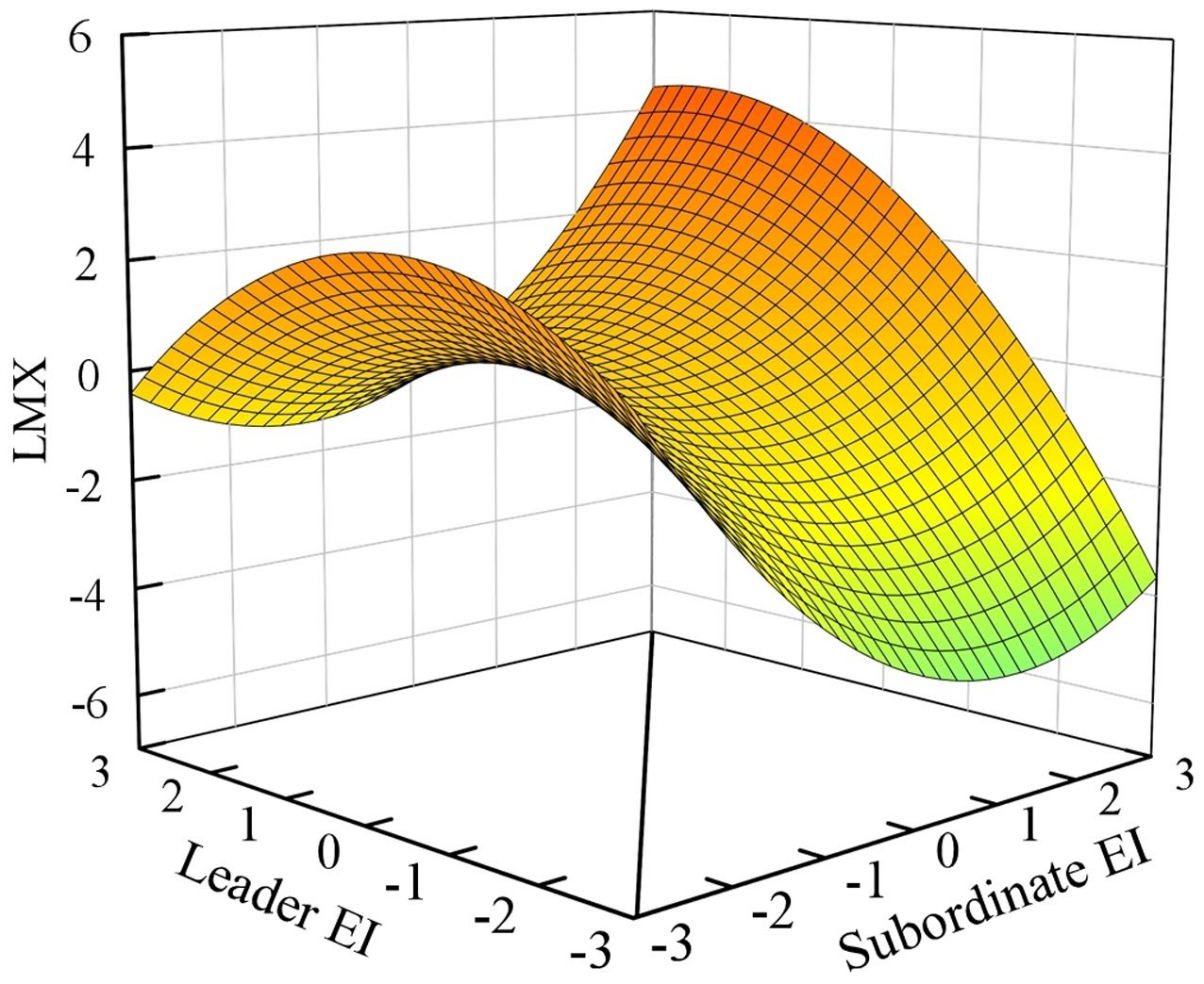
Response surface for LMX.

Hypothesis 1 examines the effect of leader-subordinate EI congruence on LMX. As shown in Table 3, the explained variance of LMX in model 3 significantly increased after including the square-item and interaction term of the leader-subordinate EI (△R^2^ = 0.22, *p* < 0.001). The significant increase in *R*^2^ indicates the existence of a non-linear association between leader-subordinate EI and LMX, providing a prerequisite for subsequent surface tests (Edwards, 2002). According to the polynomial regression of Model 3, the significant curvature along the congruence line for LMX (curvature = 0.31, *p* < 0.001) indicates that leader-subordinate EI congruence is positively related to LMX. Furthermore, I utilized mathematical software to generate a three-dimensional image for enhanced visualization of EI congruence effect. As shown in Figure 1, it can be observed that the response surface graph curves upward along the congruence line (solid line). This highlight that higher levels of leader and subordinate EI congruence (at the back top corner of Figure 1), or lower levels of leader and subordinate EI congruence (at the front top corner of Figure 1), result in the higher levels of LMX, thus providing support for the hypothesis 1. Therefore, Hypothesis 1 is supported.

**Table 3:** Results of polynomial regression analysis.

Variables	LMX			Flow experience
	Model 1	Model 2	Model 3	Model 4
Constant	−0.03	0.14	0.01	−0.41
Gender similarity	−0.04	−0.07	−0.08	0.01
Age similarity	0.00	−0.02	−0.02	0.06
Tenure with leader	0.01	−0.01	−0.01	0.00
Education similarity	0.01	0.04	0.04	0.02
EI of leader (L)		−0.08	0.01	−0.04
EI of subordinate (S)		0.64***	0.57***	0.24***
L^2^			0.32***	0.15*
L × S			0.26***	0.15*
S^2^			−0.27***	−0.17*
LMX				0.22*
△R^2^		0.38***	0.22***	0.32***
Congruence line (L = S)				
Slope			0.59***	
Curvature			0.31***	
Incongruence line (L = − S)				
Slope			−0.55***	
Curvature			−0.22**	

*** *p* < 0.001, ** *p* < 0.01, * *p* < 0.05.

EI, emotional intelligence; LMX, leader-member exchange.

Hypothesis 2 predicts that when there is congruence in EI between leaders and subordinates, a high-high matching pattern of EI leads to higher LMX compared to a low-low matching pattern. To test this hypothesis, it is necessary to consider the slope along the congruence line (solid line). The findings support our hypothesis, as they demonstrate a significant positive slope along the congruence line (slope = 0.59, *p* < 0.001). Furthermore, the response surface in Figure 1 illustrates the result: It shows that LMX is higher in the rear corner (where both leader and subordinate EI are highest) than in the front corner (where both leader and subordinate EI are lowest).

Hypothesis 3 predicts that LMX mediates the positive relationship between leader-subordinate EI congruence and flow experience. To verify the mediating effect, I initially conducted a polynomial regression analysis and introduced the mediating variable of LMX (Model 4 in Table 3). The regression coefficient for LMX was found to be significant (r = 0.22, *p* < 0.05), thereby providing preliminary evidence supporting Hypothesis 3. Furthermore, following the recommendations of Edwards and Cable (2009), the construction of a block variable was undertaken to further validate the indirect impact of leader-subordinate EI congruence on flow experience through LMX. I combined the estimated coefficients (as shown in Eq. 1) to obtain the block variable, which represents a weighted linear combination of five polynomial terms: L (leader EI), S (subordinate EI), L^2^ (leader EI square), L × S (the product of the leader EI and subordinate EI), and S^2^ (subordinate EI square). The indirect effect of EI congruence on flow experience via LMX can be calculated by multiplying the coefficient of the block variable on LMX (the α path) with the coefficient of LMX on flow experience (the *β* path). As shown in Table 4, the block variable for EI congruence has a positive relationship with LMX (path *α* = 1.00, *p* < 0.001, CI = [0.91, 1.10]), and LMX is positively associated with flow experience (path *β* = 0.22, *p* < 0.01,CI = [0.06, 0.38]). Moreover, the indirect effect through LMX was also significant and did not include zero (*α* × *β* = 0.22, *p* < 0.01, CI = [0.06, 0.39]). Thus, the overall findings provide support for hypothesis 3.

**Table 4:** Examinations of indirect effects.

Variables	Emotional intelligence (block variable) to LMX	LMX to flow experience controlling for emotional intelligence congruence	Indirect effect of LMX to flow experience
	*α* path	*β* path	*α* × *β*
Results	1.00***	0.22**	0.22**
95% bias-corrected bootstrapped CI	(0.91, 1.10)	(0.06, 0.38)	(0.06, 0,39)

*** *p* < 0.001, ** *p* < 0.01, * *p* < 0.05.

Additionally, the validity of findings is further enhanced through the implementation of a robustness check, which examines the impact of our control variables on the results (Becker, 2005). Upon removing these control variables, the conclusions drawn from this study show no significant change. Therefore, I believe that the findings of data analysis support the theories proposed in this study.

## 5 Discussion

Building upon PE-fit theory, this study conducted an examination of the complex relationship among leader-subordinate EI congruence, LMX, and flow experience. This study utilized polynomial regression and response surface analysis methods based on three-wave paired data collected from a sample of 56 leaders and 279 subordinates. The results indicate that first, leader-subordinate EI congruence is positively related to LMX. Second, a high-high EI matching pattern can enhance the favorable dynamics of congruence and yield higher LMX compared to a low-low matching pattern. Third, LMX acts as a mediator in the positive relationship between leader-subordinate EI congruence and flow. Additionally, findings in this study demonstrate that the misfit resulting from pairing a high EI subordinate with a low EI leader has a more detrimental impact on the LMX than the misfit resulting from pairing a low EI subordinate with a high EI leader. The following sections will discuss how our findings contribute to both theoretical and managerial implications in a more comprehensive manner.

### 5.1 Theoretical implications

First, this research makes two contributions to the literature on PE-fit. First, this study expands the scope of PE fit theory by specifically examining fit along a deep-level characteristic, namely EI, and present evidence that the psychological states of subordinates are influenced not only by their isolated personal characteristics but also by the level of fit between the focal leaders’ and subordinates’ characteristics. This underscores the significance of PE fit in fostering desirable psychological states among subordinates, which should not be underestimated. I highly recommend further empirical examination of PE fit in other studies on positive organizational behavior (POB) as it has the potential to generate more fascinating discoveries. Second, this study makes a valuable contribution to the existing body of knowledge on asymmetric PE-fit. As Edwards (2008) emphasized that the lack of attention to the differential effects of congruence at high and low levels is a notable limitation in PE-fit studies. Some scholars in the field argue that regardless of absolute levels, PE-fit leads to the same outcomes, while others have doubts about this proposition that require further verification (p. 216) (Edwards, 2008). Therefore, in order to advance the PE fit theory, it is imperative to explore the crucial nuances of leader-subordinate EI congruence. The slope in Table 3 indicate that the impact of congruence or incongruence effects on EI varies depending on the level of EI exhibited by both leaders and subordinates, rather than being equal positive or negative. To be more precise, a high-high EI matching pattern can enhance the favorable dynamics of congruence and yield higher desirable outcomes compared to a low-low matching (slop alone the congruence line =0.59, *p* < 0.001). Likewise, low-high leader-subordinate EI matching leads to a more negative impact than high-low matching (curvature alone the incongruence line = −0.22, *p* < 0.01; slop alone the incongruence line = −0.55, *p* < 0.001). Overall, this study responds to the call made in previous research to explore the complex nature of PE-fit (Edwards, 2008) and present novel corroborative evidence that supports the asymmetric effect in PE-fit.

Second, this study offers two contributions to the existing literature on flow. First, the flow channel model (Csikszentmihalyi and Rathunde, 1993) has already identified one person-environment fit combination as a precursor to the flow experience: specifically, the alignment between a person’s skills and task demands. Findings in this study extend the concept of fit in the flow channel model by demonstrating that leader-subordinate EI fit combination can also contribute to predicting flow, thereby offering new avenues for future research. This effectively addresses the gap highlighted in previous study (Peifer and Wolters, 2021) by providing compelling evidence that the development of flow is a multifaceted process where the interaction between leaders and subordinates should not be overlooked. Second, this study enhances the understanding of the locus of flow by examining different EI interaction scenarios, allowing for a comparison of the influence exerted by all underlying characteristics of EI at each locus (i.e., leader and subordinate). This approach facilitates the identification of influential factors in flow research at deeper levels, thereby highlighting promising areas for further study. Specifically, this study demonstrates that a high-high EI matching pattern can result in a greater flow experienced by subordinates through LMX compared to a low-low matching pattern (slope along the congruence line = 0.59, *p* < 0.001). I suggest that this may be attributed to the potential of the high-high scenario in enhancing positive dynamics of congruence compared to the low-low scenario. Therefore, future research on flow could explore environmental and individual factors that might foster favorable dynamics of congruence. To summarize, this study contributes to the advancement of flow theory within a comprehensive and inclusive framework.

Third, this study provide innovative perspectives to the current body of literature on EI and LMX. First, to date, limited attention has been given to exploring the impact of crucial nuances in leader-subordinate EI dynamics on LMX. By employing polynomial regressions and response surface analyzes, this study unveil the “black box” of asymmetric congruence effect in EI, revealing that the high-high EI congruence scenario can lead to higher levels of LMX compared to the low-low scenario. Furthermore, this study uncovers the nuanced effects of EI incongruent scenarios. The results indicate that the negative impact on LMX resulting from pairing a high EI subordinate with a low EI leader is more pronounced than the impact resulting from pairing a low EI subordinate with a high EI leader (slope along the incongruence line = −0.55, *p* < 0.001; curvature along the incongruence line = −0.22, *p* < 0.01). This finding aligns with previous research (Marstand et al., 2017), which suggests that LMX was found to be lowest when the leader did not fulfill subordinate’s high work values. Thus, the present study builds upon existing research on the impact of EI on the development of social interaction (Lopes et al., 2004) by comprehensively examining the crucial role that EI congruence and incongruence can play in fostering leader-subordinate interactions, particularly LMX. Second, this study not only aligns with previous research findings that indicate a significantly positive impact of leader-subordinate fit on LMX (van Vianen et al., 2011), but also expands upon the insights provided by Sy et al. (2006) by suggesting that even when there is a lower level of EI congruence, leader-subordinate congruence can still result in comparatively high LMX.

### 5.2 Managerial implications

Based on the results of this study, two managerial implications are proposed: First, findings in this study highlight the significance of leader-subordinate EI congruence in shaping the focal subordinate’s flow experience. Therefore, in order to enhance subordinates’ flow, organizations should allocate time and resources not only to the development of leaders’ and subordinates’ EI, but also to improving EI congruence through strategic team configuration, particularly by ensuring high-high EI matching.

Second, if there is an unavoidable incongruence in EI between leaders and subordinates, the findings suggest that interventions focused on enhancing LMX may yield advantageous outcomes. As highlighted by Edwards and Cable (2009), addressing mediating mechanisms can effectively compensate for a lack of PE fit. Given the crucial role of communication in LMX (Graen and Uhl-Bien, 1995), leaders could reap some benefits of EI congruence through regular and transparent conversations. In addition, previous studies indicate that leadership training effectively enhances communication skills (Frese et al., 2003). Therefore, this study highly recommends that organizations provide training for managers to adjust their communication styles in order to enhance subordinates’ positive perceptions.

### 5.3 Limitations and future research

The present study adhered to the principles of scientific research and yielded intriguing findings; however, it is important to acknowledge several limitations. First, despite collecting data from multiple sources at three different time points with one-month intervals to minimize the potential impact of CMV, the research design primarily relied on self-reported questionnaires. Therefore, further investigation is needed to integrate questionnaire surveys, experimental designs, and diary studies in order to comprehensively examine the implied causal flow in my model.

Second, the data were collected from the liquor manufacturing industry in China, so it is important to acknowledge that these findings may not fully represent other industries or cultural contexts. Therefore, this study suggests that future research should replicate this methodology in various cultural and industrial contexts to test the generalizability of the findings and identify any culturally and industrially specific aspects. This approach may enhance the external validity of this study and potentially uncover additional fascinating results.

Third, although the current study confirms the significance of leader-subordinate EI congruence on subordinates’ flow experience, it is strongly recommended that future research incorporates group flow, a collective state that occurs when a group is performing at the peak of its abilities (Sawyer, 2003), into my model to explore and identify intriguing findings related to flow experience at different levels. In addition, other dimensions of leader-subordinate interaction, such as power distance orientation interaction, may also yield intriguing insights.

## Data availability statement

The raw data supporting the conclusions of this article will be made available by the authors, without undue reservation.

## Ethics statement

Ethical review and approval was not required for the study on human participants in accordance with the local legislation and institutional requirements. Written informed consent from the patients/participants or patients/participants legal guardian/next of kin was not required to participate in this study in accordance with the national legislation and the institutional requirements.

## Author contributions

ZL: Writing – review & editing, Writing – original draft.
